# Stirring time effect of silver nanoparticles prepared in glutathione mediated by green method

**DOI:** 10.1186/1752-153X-8-11

**Published:** 2014-02-13

**Authors:** Sepideh Keshan Balavandy, Kamyar Shameli, Dayang Radiah Binti Awang Biak, Zurina Zainal Abidin

**Affiliations:** 1 Institutes of Advanced Technology, Universiti Putra Malaysia, 43400 UPM Serdang, Selangor, Malaysia; 2 Department of Chemistry, Faculty of Science, Universiti Putra Malaysia, 43400 UPM Serdang, Selangor, Malaysia; 3 Nanotechnology and Advance Materials Department, Materials & Energy Research Center, 31787/316 Alborz, Karaj, Iran; 4 Department of Chemical and Environmental Engineering, Faculty of Engineering, University Putra Malaysia, 43400 UPM Serdang, Selangor, Malaysia

**Keywords:** Silver nanoparticles, Green chemistry, Glutathione, Stirring time, Transmission electron microscopy

## Abstract

**Background:**

This study aims to investigate the influence of different stirring time for synthesis of silver nanoparticles in glutathione (GSH) aqueous solution. The silver nanoparticles (Ag-NPs) were prepared by green synthesis method using GSH as reducing agent and stabilizer, under moderate temperature at different stirring times. Silver nitrate (AgNO_3_) was taken as the metal precursor while Ag-NPs were prepared in the over reaction time.

**Results:**

Formation of Ag-NPs was determined by UV–vis spectroscopy where surface plasmon absorption maxima can be observed at 344–354 nm from the UV–vis spectrum. The synthesized nanoparticles were also characterized by X-ray diffraction (XRD). The peaks in the XRD pattern confirmed that the Ag-NPs possessed a face-centered cubic and peaks of contaminated crystalline phases were unable to be located. Transmission electron microscopy (TEM) revealed that Ag-NPs synthesized were in spherical shape. Zeta potential results indicate that the stability of the Ag-NPs is increases at the 72 h stirring time of reaction comparison to GSH. The Fourier transform infrared (FT-IR) spectrum suggested the complexation present between GSH and Ag-NPs. The use of green chemistry reagents, such as peptide, provides green and economic features to this work.

**Conclusions:**

Ag-NPs were successfully synthesized in GSH aqueous solution under moderate temperature at different stirring times. The study clearly showed that the Ag-NPs synthesized in the long times of stirring, thus, the kinetic of GSH reaction is very slow. TEM results shows that with the increase of stirring times the mean particle size of Ag-NPs become increases. The FT-IR spectrum suggested the complexation present between GSH and Ag-NPs. These suggest that Ag-NPs can be employed as an effective bacteria inhibitor and can be applied in medical field.

## Background

Nanotechnology and especially nanomaterials have received much consideration because their structure and properties differ appreciably from those of molecules, atoms, and bulk materials [1]. The synthesis of metal nanoparticles has been widely discussed in the literature due to their distinctive chemical and physical properties, which have many potential purposes [2,3]. The utilization of non-toxic solvents, biodegradable materials and low-cost green chemicals are central to resources synthesis and processing, considering the green reaction method of these strategies. The stabilizer, reaction medium, and green reducing agent are three key factors in the synthesis and stabilization of metallic nanoparticles [4].

Due to its properties and areas of use, Ag is one of the most studied metals. Stability, morphology, particle size distribution and surface state charge/modification, all play a very significant role and there is much interest in the controlled synthesis of Ag-NPs. The literature describing the preparation of Ag-NPs is particularly broad since the classical colloid methods are combined with modern nanotechnology leading to many procedures for surface modification, particle size control, particle preparation [5]. Many synthesis methods have been applied to prepare Ag-NPs; chemical reduction in aqueous and non-aqueous media, in soft matrices, and in solid matrices (e.g., mesoporous silicate) [6-8], by applying physical processes/various types of irradiation [2,9], and electrochemical processes [10], in emulsion systems [11]. The Ag-NPs are widely used as photo-catalysts [12], catalysts [13], antibacterial [14], biosensor [15], bioimaging [16] and in plant extracts [17,18].

The choice of glutathione (GSH), as reducing agent and stabilizer were made because of its benign nature and the presence of a highly reactive thiol group that can be used to reduce the metal salts. GHS is a tripeptide consisting of glutamic acid, cysteine and glycine units and is a ubiquitous antioxidant present in human and plant cells. Besides the thiol group, each GSH molecule also contains amine and carboxylate functionalities that provide coupling possibilities for further cross-linking to other molecules of biological or sensing interest [19]. The surface passivation reagents, including functional groups in molecules, are needed to prevent the nanoparticles from aggregation. The GSH has been widely applied as an effective passivation agent in the fabrication of Ag-NPs and other metal nanoparticles. Recent reports revealed that GSH was effective for the control of size and shape of Ag NPs. The surface modification of these colloidal nanoparticles is very important to facilitate their application to biotechnology and nanocomposites [19,20].

The synthesis of Ag-NPs in the mineral compounds and/or organic polymer substrates for example in the talc [21], montmorillonite [22,23], starch [24], chitosan [25], polyethylene glycol [26,27], poly(lactic acid) [28], and/or plant extracts [29-32], were studies according to our previous work. The synthesis method presented could be useful in providing an economic method for the preparation of compatible, and stable colloidal Ag-NPs. After 36, 48 and 72 h from reaction times, the nanoparticles started for synthesis and mean particle size gradually increases with less than 7 nm. Other benefits of this method contain the physical conditions of the synthesis, such as the use of atmospheric pressure, the lack of need for an additional flow of inert gas and the moderate reaction temperature.

## Results and discussion

**Figure 1:**
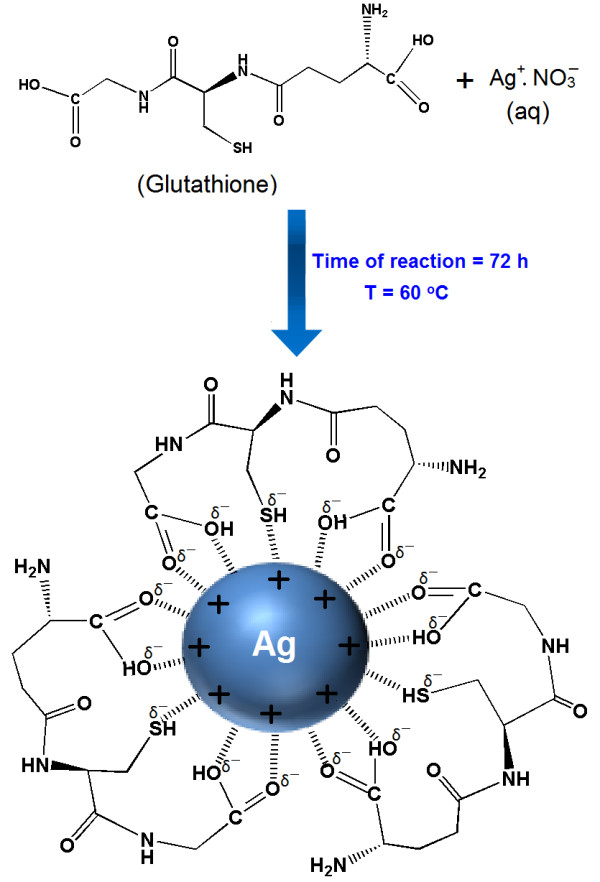
Schematic illustration showing the interactions between partial negative charges of thiol, and hydroxyl groups of GSH structures with surface positive charges of Ag-NPs.

In this research, GSH were appropriate as a green reducing agent and polymeric stabilizer media for reducing the AgNO_3_ in moderated temperature (60°C). The schematic illustration of the synthesis of Ag-NPs capped with GSH is depicted schematically in Figure 1.

As shown thiol (−SH) and acetyl (−COO) groups of GSH as a capping agent can form week bonds with the surface of Ag-NPs. This is due to the surface of Ag-NPs which is positively charge. Certainly, we suppose that colloidal stabilization for [Ag(GSH)] occur due to the presence of van der waals forces between the negatively partial charges of thiol and acetyl groups that present in the molecular structure of the GSH, with positive charge of Ag-NPs that surround on the surface of NPs. Figure 1 illustrates the interaction between the charged Ag-NPs and GSH [19].

The GSH reduce silver ions to Ag-NPs, also through this process GSH have size controller and capping agent roles. The possible chemical equations for preparing the Ag-NPs are:

(1) $${{\text{Ag}}^{+}}_{\left(\text{aq}\right)}+{\text{GSH}}_{\left(\text{aq}\right)}\to {{\left[\text{Ag}\left(\text{GSH}\right)\right]}^{+}}_{\left(\text{aq}\right)}$$

(2) $${{\left[\text{Ag}\left(\text{GSH}\right)\right]}^{+}}_{\left(\text{aq}\right)}\overset{T=60\;{}^{\circ}C,\;t=72\;h0}{\underset{\left(+1e\right)}{\to }}\;\left[\text{Ag}\left(\text{GSH}\right)\right]↓$$

After dispersion of silver ions in the aqueous solution of GSH matrix (Equation 1), GSH reacted with the Ag^+^ to form a GSH complex [Ag(GSH)]^+^, which reacted to form [Ag (GSH)] (Equation 2).

**Figure 2:**
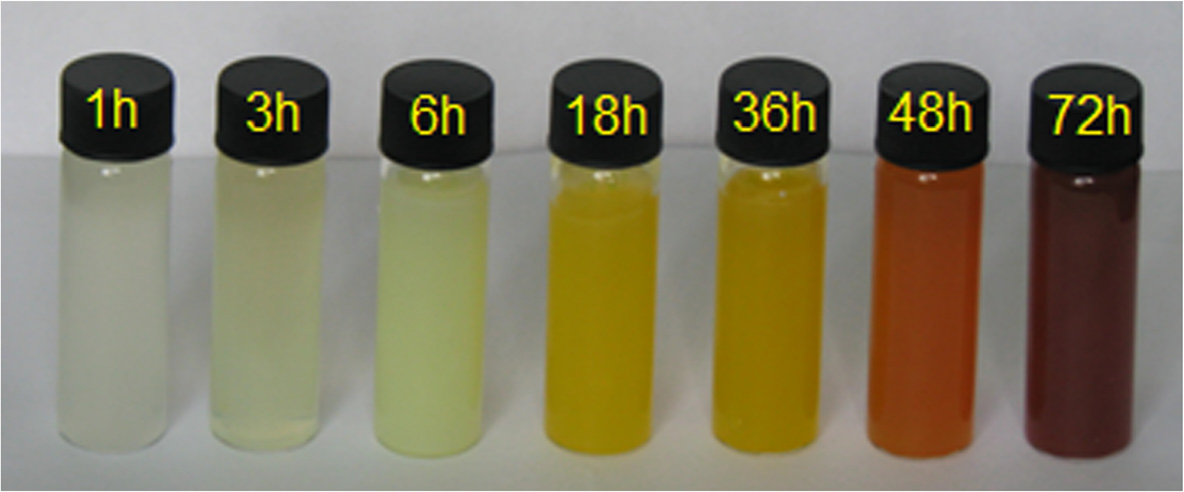
Photograph of Ag-NPs prepared at different times of reaction in GSH in the modetare temperature for 1, 3, 6, 18, 36, 48, 72 h, respectively.

The colorless solution turned to the pale yellow, after 1, 3 and 6 h, indicating the initial formation of Ag-NPs. When we increased time of the reaction, the color changed to yellow after 18 and 36 h, while with continuous stirring at a moderate temperature for 48 h, the color of the reaction changed to light brown. Also, with the increase time of reaction to 72 h, the color of the reaction changed to dark brown. These observations show that with an increase in reaction time, particle size and aggregation of silver nanocrystal gradually increased together. Herein GSH eventually plays a multifunctional role in the synthesis of the Ag-NPs. This antioxidant successfully reduces silver nitrite during the 72 h in the 60°C (Figure 2).

### UV–visible spectroscopy

**Figure 3:**
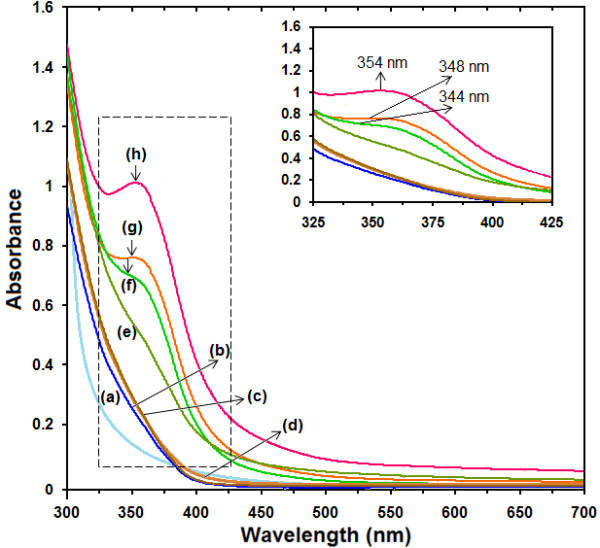
**The UV–visible spectrum of Ag**^**+ **^**and Ag-NPs prepared in GSH solution at different stirring times [0, 1, 3, 6, 18, 36, 48 and 72 h (a–h)].**

The formation of Ag-NPs in the GSH media was further determined by using the UV–visible spectroscopy, which was shown on the surface plasmon resonance (SPR) bands. Figure 3 shows that Ag-NPs started forming when [Ag(GSH)]^+^ reacted at a moderate temperature. The [Ag(GSH)]^+^ peak was not observed at the beginning of the reaction. Moreover, with the increase reaction time to 1, 3, 6, and 18 h, the absorbance peaks could not see in GSH substrate.

Generally, the SPR bands are influenced by the size, shape, morphology, composition and dielectric environment of the prepared nanoparticles [33,34]. Previous studies have shown that the spherical Ag-NPs contribute to the absorption bands at around 350 nm in the UV–visible spectra [35]. From this research, the SPR band characteristics of Ag-NPs were detected around 344–354 nm [Figure 3f‒h], which strongly suggests that the Ag-NPs were spherical in shape and have been confirmed by the TEM results of this study. As shown, when the stirring time of reaction was increased, the intensity of the SPR peak also gradual increase until 72 h. Therefore this shows that the reduction of the silver ions to silver atoms continued and resulted in an increase in the concentration of Ag-NPs.

Thus, there is a normal case in this situation for the SPR absorption band for the particles, which agreed with the TEM results, whereby red–shifts were observed as size increased in the during the reaction after 36, 48 and 72 h, respectively. This can be explained by the multilayer Mie theory model, which theorizes that the chemical interaction caused the lowered electron conductivity in the outermost atomic layer and consequently caused the red–shifts [36].

As seen from the Figure 3h, it can be observed that 72 h had large absorbance compared to 48, 36 h because the particle size of Ag-NPs after 72 h were larger than those at 48, 36 h. Also, absorption spectra of larger metal colloidal dispersions can exhibit broad peaks. This phenomenon could be due to the fact that, after reaching a certain particle size, the GSH as stabilizer was able to withhold the nanoparticle’s size effectively.

### Powder X–ray diffraction

**Figure 4:**
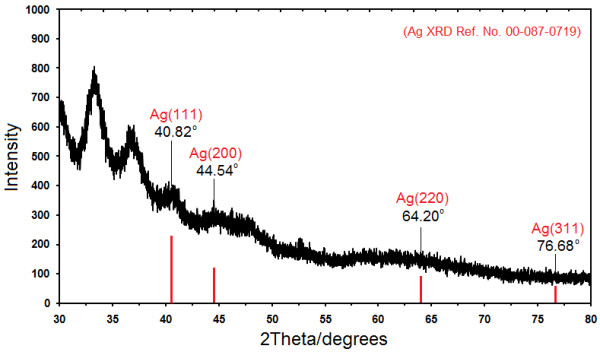
X-ray diffraction patterns of Ag-NPs synthesized in GSH after 72 h stirring time of reaction.

Figure 4 shows the XRD patterns of Ag-NPs formed in the 72 h from stirring time of reaction, which indicates the formation of the silver crystalline structure face-centered cubic. Moreover, all the Ag-NPs had a similar diffraction profile, and XRD peaks in the wide angle range of 2θ (30° < 2θ < 80°) point out that the peaks in 40.82°, 44.54°, 64.20°, and 76.68° could be attributed to the 111, 200, 220, and 311 crystallographic planes of the face-centered cubic silver crystals, respectively [37,38]. The XRD pattern thus clearly illustrated that the Ag-NPs formed in this study were crystalline in nature. The main crystalline phase was silver, and there were no obvious other phases as impurities were found in the XRD patterns [39,40].

The average particle size of silver nanoparticles can be calculated using Debye–Scherrer Equation (3):

(3) $$n=\frac{\text{Kλ}}{\beta \text{cosθ}}$$

Where K is the Scherrer constant with value from 0.9 to 1 (shape factor), where λ is the X-ray wavelength (1.5418 Å), β_1/2_ is the width of the XRD peak at half height and θ is the Bragg angle. From the Scherrer equation the average crystallite size of Ag-NPs for 36, 48 and 72 h times of reaction are found to be around 3–8 nm, which are also in line with the observation of the TEM results discussed later.

### Morphology study

**Figure 5:**
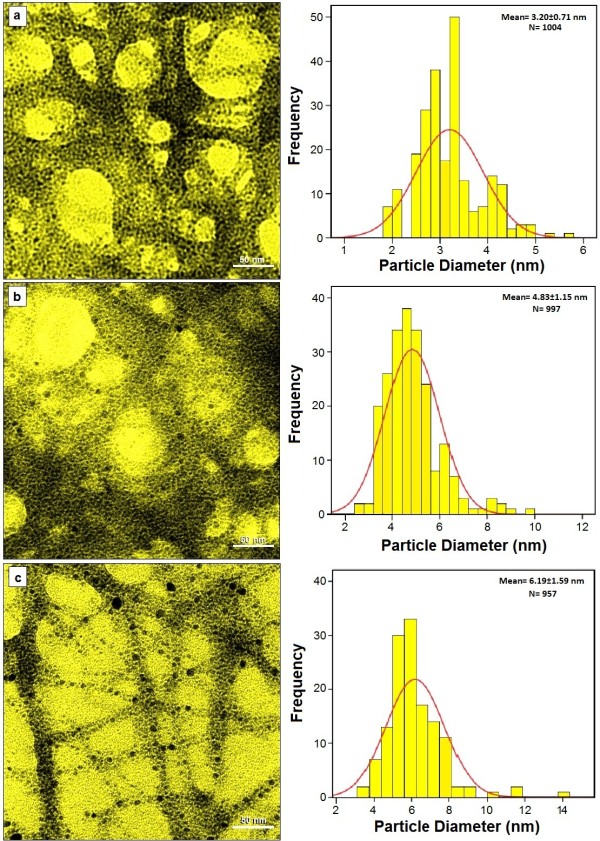
Transmission electron microscopy image and particle size distribution of Ag-NPs after 36, 48 and 72 h of stirring times, (a–c) respectively.

The TEM images and their corresponding particle size distributions of Ag-NPs at different periods of time are shown in Figure 5. The TEM images and their size distributions revealed that, the mean diameters and standard deviation of Ag-NPs were about 3.20 ± 0.71, 4.83 ± 1.15 and 6.19 ± 1.59 nm for 36, 48 and 72 h (a–c), respectively. The total numbers of Ag-NPs counted for each TEM images were about 1004, 997 and 957 for 36, 48 and 72 h, respectively. These results approved that with increase in time of reaction at a moderate temperature, mean diameters and standard deviations of the Ag-NPs gradually increases.

The presences of one narrow distribution of Ag-NPs in TEM image are in accordance with the UV–Vis spectral study. Figure 5a‒c show the Ag-NPs surrounded by the extract of GSH. The dark points in this figure represent the large scale distribution of Ag-NPs. The Ag-NPs surrounded by GSH is shown by TEM in Figure 5 and confirmed by FT‒IR spectroscopy.

**Figure 6:**
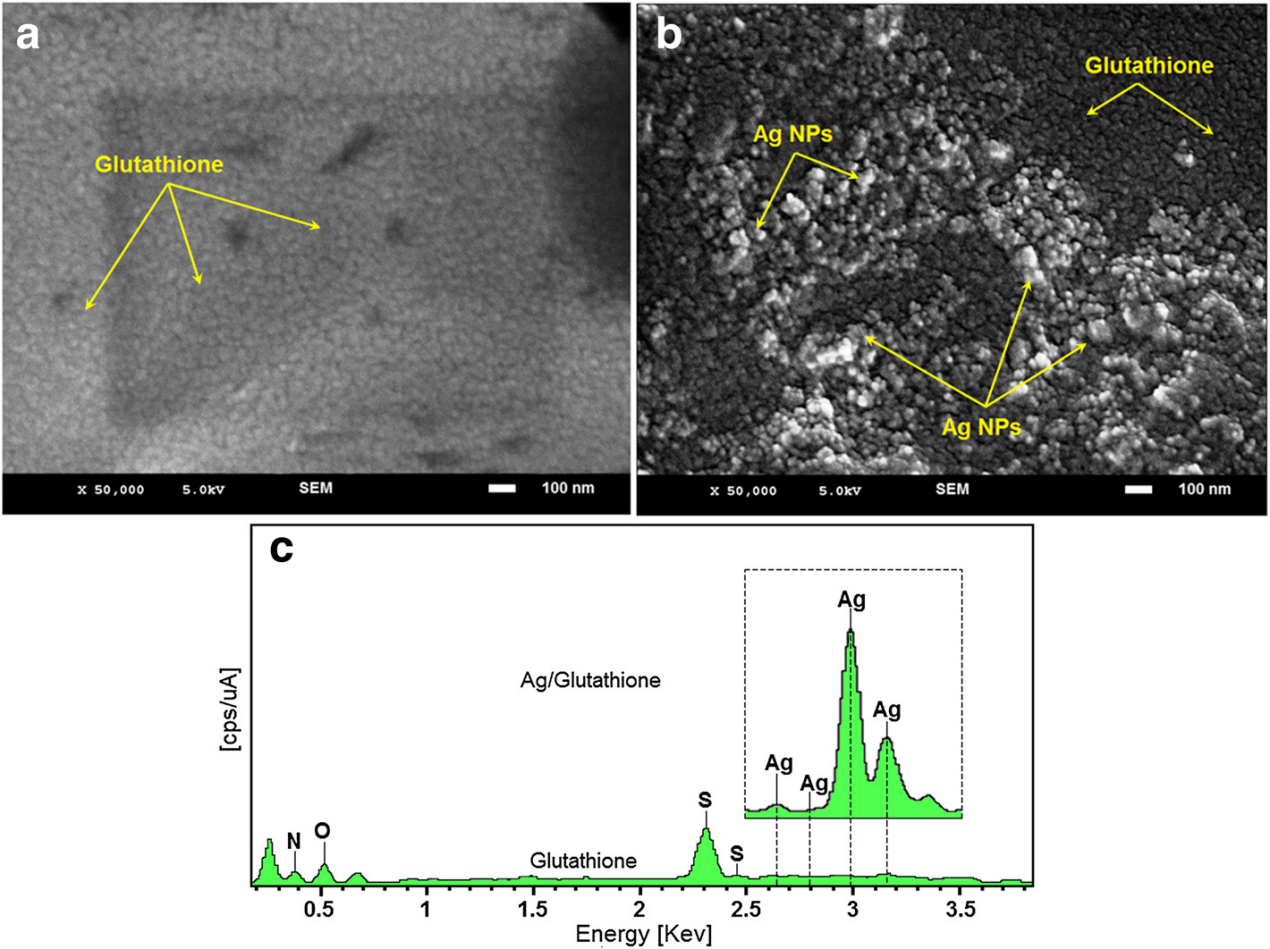
SEM image and EDXRF spectra of GSH and [Ag(GSH)] formation after 72 h of stirring time respectively (a–c).

The Figure 6a‒c shows the SEM images and EDXRF spectrum for the GSH and [Ag (GSH)] after 72 h from stirring time [Figure 6a‒b]. These results confirm that GSH can effectively control shape and size of the Ag NPs after 72 h in moderate temperature.

The exterior surfaces of [Ag (GSH)] due to the presence of small size Ag-NPs become shiny in the spots spherical shapes. Figure 6c, shows the EDXRF spectra for the GSH and [Ag (GSH)] after 72 h; the peaks around 0.72, 2.30 and 2.45 keV are related to the binding energies of GSH [41]. The peaks around 2.65, 2.80, 3.00 and 3.16 keV are related to the silver elements in the GSH (Figure 6c).

### Zeta potential measurement

**Figure 7:**
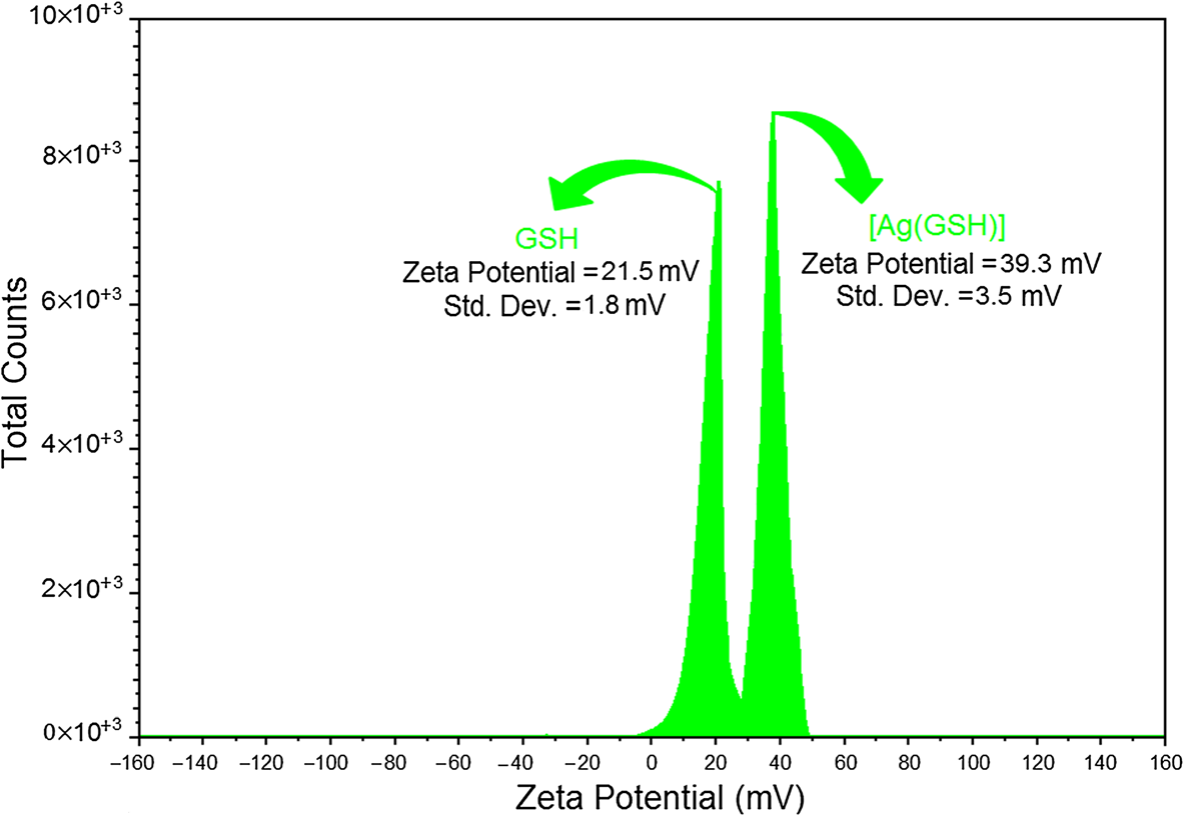
Zeta potential for GSH and [Ag(GSH)] aqueous solution after 72 h from stirring times.

As shown in the Figure 7, the Ag-NPs obtained possess a positive zeta potential value. Zeta potential is an essential parameter for characterization of stability in aqueous Ag-NPs suspensions. A minimum of ±30 mV zeta potential values is required for indication of stable nano-suspension [42-44]. At the GSH and [Ag (GSH)] after 72 h of stirring time zeta potential were equals to 21.5 ± 1.8 and 39.3 ± 3.5 mV. So, this result clearly indicate that the particles are fairly stable at the 72 h stirring time of reaction, moreover, with the change of zeta potential from GSH to [Ag (GSH)] the stability increased.

### FT-IR chemical analysis

**Figure 8:**
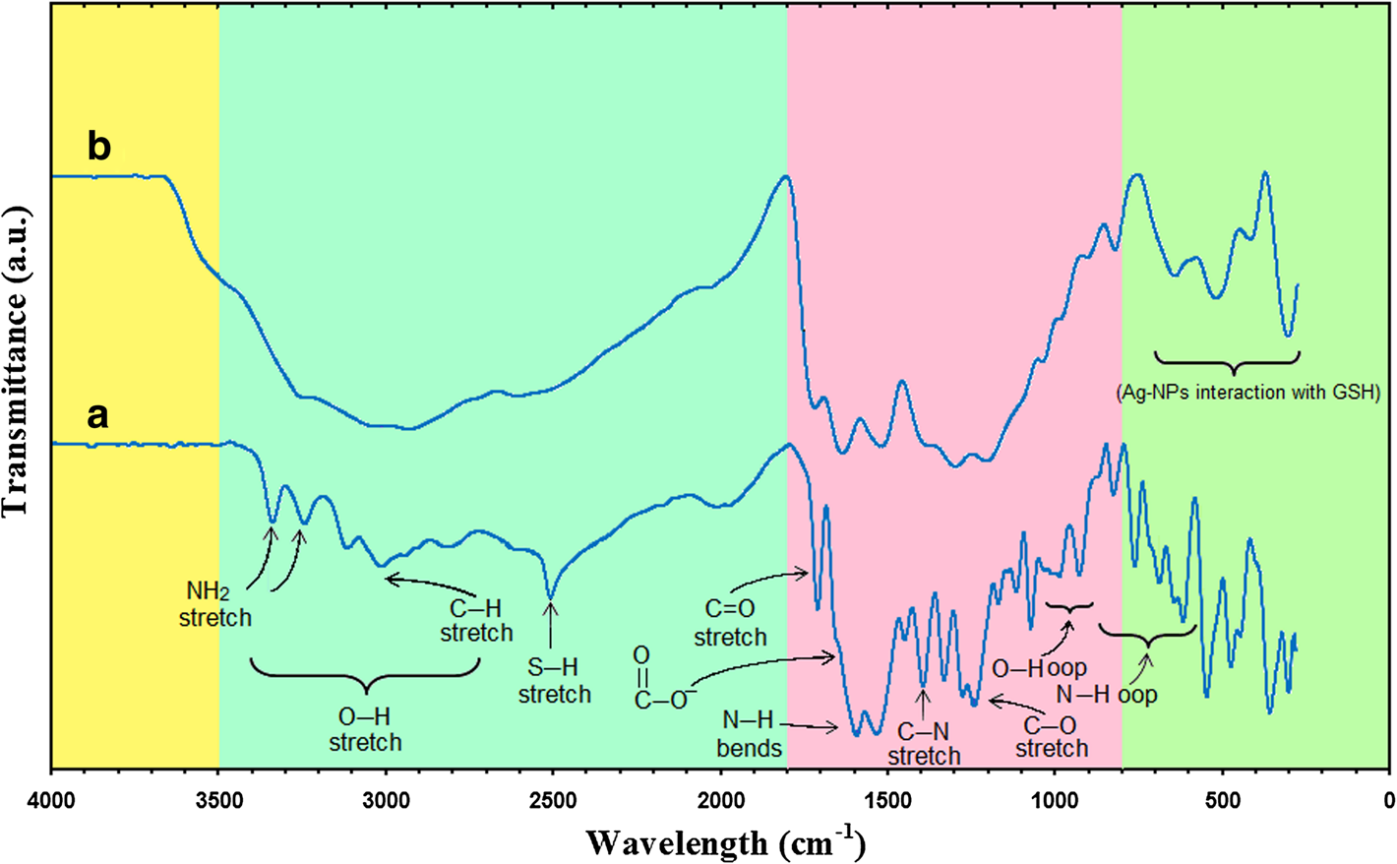
Fourier transform infrared spectra for GSH (a), [Ag (GSH)] after 72 h (b) from stirring time.

The interactions of Ag-NPs obtained with GSH were confirmed by FT-IR spectra. Figure 8 shows the FT-IR spectra of pure GSH and [Ag (GSH)]. Comparing the FT-IR spectra of pure GSH and [Ag (GSH)], significant features can be seen: the characteristic absorption peaks for S‒H and N‒H stretching bands at 2510, 3331 and 3234 cm^−1^ as found in pure GSH have disappeared in [Ag (GSH)]. The dramatic differences between the FT-IR spectra, especially for S‒H and N‒H, suggest that GSH is modified onto the surface of Ag-NPs via the thiol and amine groups from the cysteine moiety of GSH. It is perfectly in agreement with the reported literatures [45].

On the other hand, as for the GSH spectrum, the absorption bands at around 3000 cm^−1^ were due to the O‒H stretching band, 2900 cm^−1^ was due to the aliphatic C‒H stretching, 1441, 1388 and 1324 cm^−1^ were due to C‒H bending vibrations, and also the combination band of O–C‒H and C‒O‒H deformation is calculated from 1441 to 1324 cm^−1^. Then the out of plane (oop) C‒H and N‒H deformation from 1231 to 920 cm^−1^ and 819 to 611 can be observed. The region from 1163 to 540 cm^−1^ contains C‒O and C‒C groups’ vibration modes are present and the carboxylic groups that generally shows their characteristic bands [43]. The bands due to C–O stretching mode got merged in the broad envelope centered on 1231 and 1068 cm^−1^ arising from C–O, C–O–C stretches and C–O–H bends vibrations of Ag-NPs in GSH. The bond peak due to C‒N stretching appeared around 1388 cm^−1^ that related to amine and amides groups.

After the bio-reaction of AgNO_3_ in the GSH matrix, the created peak in 1705 cm^−1^ certified to the binding of ‒C = O for carboxylic acid, and the shift in the peak at 1632 cm^−1^ towards lower frequency compared to peak in 1705 cm^−1^ for GSH. The broad peaks from 639 to 299 cm^−1^ are related to Ag-NPs banding with sulfur from thiol groups of GSH molecules.

Thus, as shown thiol group of GSH as capping agent can make a cover in the surface of Ag-NPs. This is possible because the surface of Ag-NPs is positively charged. Certainly, we suppose that colloidal stabilization for [Ag(GSH)] occur due to the presence of van der waals forces between the sulfur negatively charged groups present in the molecular structure of the GSH, and the positively charged that surround the surface of the inert Ag-NPs [46]. Therefore, the FT-IR spectra showed the existence molecular interactions between the Ag-NPs with the tripeptide media [47].

## Conclusion

In summary, we have described a simple and green method of colloidal Ag-NPs synthesis by using green reducing agents which requires no special physical conditions. Ag-NPs were successfully synthesized under moderate temperature (60°C) at different stirring times of reaction. The formation of Ag-NPs was confirmed in the UV-visible absorption spectra, which showed the SPR band characteristics of Ag-NPs in the range of 344–354 nm. The XRD results confirmed that the Ag-NPs possessed a face-centered cubic crystal structure (fcc). In addition, this also revealed that Ag-NPs were the main composition present in the nanocomposites without any contamination peaks. The TEM images showed that the Ag-NPs were in spherical shape and the average diameters of the particles were 3.20, 4.83 and 6.19 nm for the stirring times of 36, 48 and 72 h, respectively. FT-IR spectrum suggested the complexation present between GSH and Ag-NPs to form metallopolymer [Ag (GSH)] and the stability of the Ag-NPs was confirmed with the zeta potential measurements. Needless to say, further studies are required to investigate the biological effects of [Ag (GSH)] suspension on the types of bacteria for potential widening of this subject area.

## Methods

### Materials

All reagents in this effort were analytical grade and were used as received without further purification. AgNO_3_ (99.98%) was used as a silver precursor, and was provided by Merck, Germany. The glutathione (GSH, 98%) for analysis was used as a green reducing and stabilizing of silver ions to Ag atoms and was obtained from ACROS Chemical New jersey, USA. All solutions were freshly prepared using double distilled water and kept in the dark to avoid any photochemical reactions. All glassware used in experimental procedures was cleaned in a fresh solution of HNO_3_/HCl (3:1, v/v), washed thoroughly with double distilled water, and dried before use.

#### Synthesis of Ag-NPs by glutathione using green methods

For the synthesis of Ag-NPs in glutathione (GSH), soluble of GSH (1.5 wt%) was prepared by solubilize in 50 mL double-distilled water under stirring. The aqueous solution of GSH was added to 50 mL of AgNO_3_ (0.1 M) solution under constant stirring for 72 h in 60°C. The colloid suspensions were collected in different times (1, 3, 6, 18, 36, 48 and 72 h).

#### Synthesis of Ag-NPs by using green method

The preparation of Ag-NPs in the GSH solution matrix is quite directly forward. In a typical synthesis, 50 mL of AgNO_3_ solution (0.1 M) was added to 50 mL of 1.5 wt% GSH solution to obtain the clear complex aqueous solution of [Ag (GSH)]^+^. The solutions obtained were distributed into seven cuvettes, and the prepared solutions were stirred and maintained for different periods of time, respectively [1, 3, 6, 18, 36, 48 and 72 h (a‒g)]. Throughout, the reduction processes for all solutions were kept at a temperature of 60°C in the dark place to avoid any photochemical reactions. The mixtures were heated to 60°C and maintained at this temperature for 1, 3, 6, 18, 36, 48 and 72 h (a–g) respectively. The obtained colloidal suspensions of [Ag (GSH)] were then centrifuged at 20,000 rpm for 15 min, these precipitates were washed three times using double distilled water in order to remove the silver ion residue and dried overnight at 40°C under a vacuum.

#### Characterization methods and instruments

The prepared Ag-NPs were characterized using X-ray diffraction (XRD), ultraviolet–visible spectroscopy, transmission electron microscopy (TEM), scanning electron microscopy (SEM), energy dispersive X-ray (EDX) spectroscopy and Fourier transform infrared (FT-IR). The XRD patterns were recorded at a scan speed of 2° min^−1^. Meanwhile, the structures of the produced Ag-NPs were examined using Shimadzu PXRD-6000, powder x-ray diffraction. Moreover, TEM observations were carried out using the Hitachi H-7100 electron microscopy, whereas the particle size distributions were determined using the UTHSCSA Image Tool software (Version 3.00). To ensure the formation of Ag-NPs, the colloids solutions were tested for their optical absorption property using a Shimadzu H.UV, 1650 PC UV-visible spectrophotometer over the range of 300 to 700 nm. Furthermore, SEM and EDX were performed utilizing the XL 30 Philips instrument to study the morphology of [Ag (GSH)]. Moreover, the FT-IR spectra were recorded over the range of 200–4000 cm^−1^ utilizing the Series 100 PerkinElmer FT-IR 1650 spectrophotometer. After the reactions, the samples were centrifuged by using a high-speed centrifuge machine (Avanti J25, Beckman).

## Competing interests

The authors declare that they have no competing interests.

## Authors’ contributions

S. K. Balavandy and Z. Zainal Abidin carried out the synthesis, and characterization of the compounds. K. Shameli carried out the acquisition of data, analysis and interpretation of data collected and involved in drafting of manuscript, revision of draft for important intellectual content and give final approval of the version to be published. All authors read and approved the final manuscript.
